# Automatic Evaluation Stimuli – The Most Frequently Used Words to Describe Physical Activity and the Pleasantness of Physical Activity

**DOI:** 10.3389/fpsyg.2016.01277

**Published:** 2016-08-23

**Authors:** Amanda L. Rebar, Stephanie Schoeppe, Stephanie J. Alley, Camille E. Short, James A. Dimmock, Ben Jackson, David E. Conroy, Ryan E. Rhodes, Corneel Vandelanotte

**Affiliations:** ^1^Physical Activity Research Group, School of Human, Health, and Social Sciences, Central Queensland University, RockhamptonQLD, Australia; ^2^Health Psychology and Behavioural Medicine Research Group, Faculty of Health Sciences, School of Psychology and Speech Pathology, Curtin University, PerthWA, Australia; ^3^Faculty of Health Sciences, South Australian Health and Medical Research Institute, The University of Adelaide, AdelaideSA, Australia; ^4^School of Sport Science, Exercise, and Health, The University of Western Australia, PerthWA, Australia; ^5^Department of Kinesiology and Human Development and Family Studies, The Pennsylvania State University, University ParkPA, USA; ^6^Department of Preventive Medicine, Northwestern University, ChicagoIL, USA; ^7^Behavioural Medicine Laboratory, School of Exercise Science, University of Victoria, VictoriaBC, Canada

**Keywords:** dual process, implicit, non-conscious, modes, exercise

## Abstract

Physical activity is partially regulated by non-conscious processes including automatic evaluations – the spontaneous affective reactions we have to physical activity that lead us to approach or avoid physical activity opportunities. A sound understanding of which words best represent the concepts of *physical activity* and *pleasantness* (as associated with physical activity) is needed to improve the measurement of automatic evaluations and related constructs (e.g., automatic self-schemas, attentional biases). The first aim of this study was to establish population-level evidence of the most common word stimuli for *physical activity* and *pleasantness*. Given that response latency measures have been applied to assess automatic evaluations of physical activity and exercise, the second aim was to determine whether people use the same behavior and pleasant descriptors for *physical activity* and *exercise.* Australian adults (*N* = 1,318; 54.3% women; 48.9% aged 55 years or older) were randomly assigned to one of two groups, through a computer-generated 1:1 ratio allocation, to be asked to list either five behaviors and pleasant descriptors of *physical activity* (*n* = 686) or of *exercise* (*n* = 632). The words were independently coded twice as to whether they were novel words or the same as another (i.e., same stem or same meaning). Intercoder reliability varied between moderate and strong (agreement = 50.1 to 97.8%; κ = 0.48 to 0.82). A list of the 20 most common behavior and pleasantness words were established based on how many people reported them, weighted by the ranking (1–5) people gave them. The words people described as *physical activity* were mostly the same as those people used to describe *exercise*. The most common behavior words were ‘walking,’ ‘running,’ ‘swimming,’ ‘bike riding,’ and ‘gardening’; and the most common pleasant descriptor words were ‘relaxing,’ ‘happiness,’ ‘enjoyment,’ ‘exhilarating,’ ‘exhausting,’ and ‘good.’ These sets of stimuli can be utilized as resources for response latency measurement tasks of automatic evaluations and for tools to enhance automatic evaluations of physical activity in evaluative conditioning tasks.

## Introduction

Regular physical activity is essential for maintaining good physical and mental health (Warburton et al., 2006; Sattelmair et al., 2011; Rebar et al., 2015b); however, most people are not regularly physically active enough to obtain substantial health benefits (Bauman et al., 2009; Australian Bureau of Statistics, 2015). It is widely accepted that enhancing a person’s motivation can increase how active they are (Michie et al., 2009), but physical activity promotion efforts have narrowly targeted strategies meant to enhance reflective, intentional motivation such as self-monitoring and goal-setting at the expense of more non-conscious, impulsive predictors of behavior (Marteau et al., 2012; Sheeran et al., 2013). We can expand our knowledge of, and ability to promote, physical activity by focusing on the development of empirically sound tools for measuring and enhancing the non-conscious regulatory processes that also regulate physical activity behavior (Rebar et al., 2016).

Based on dual process theories, decisions to be active are the result, not only of reflective processes, which are slow and deliberate, but also non-conscious processes, which are rapid and spontaneous (Chaiken and Trope, 1999; Evans and Frankish, 2009). When opportunities to be physically active arrive, immediately (within 0.25 s!), we are biased to approach or avoid that opportunity depending on the non-conscious process referred to as ‘automatic evaluations’. *Automatic evaluations* are the immediate affective (i.e., pleasant/unpleasant) responses a person has toward an event or stimulus, which go on to influence decisions and behaviors (Murphy and Zajonc, 1993; Bargh et al., 1996; Cunningham et al., 2004). Although the exact origin of a person’s automatic evaluations is still unknown, it is suspected that they are an amalgamation of experiences with the event/stimulus and of the concepts and beliefs that they associate with it (Rudman, 2004; Cunningham et al., 2007).

Importantly, automatic evaluations may or may not be consistent with evaluations that a person reports about a behavior after some reflection. For example, a person could have somewhat unpleasant automatic evaluations of physical activity but self-report having strongly pleasant evaluations after some reflection. The exact interplay between non-conscious and reflective evaluations and their impact of physical activity behavior remains unclear, but evidence suggests that they are distinct (Hyde et al., 2010) and have distinct influences over a person’s physical activity behavior (Conroy et al., 2010).

People who automatically associate physical activity cues (e.g., words, images) with the concept of pleasantness are more physically active than people who do not have these associations – one study showed that 14% of physical activity behavior can be explained by these automatic evaluations (Rebar et al., 2015a). Automatic evaluations of physical activity have been assessed with a variety of response latency measures like the Implicit Association Test (Greenwald et al., 1998) or the Extrinsic Affective Simon Task (De Houwer, 2003). Although the procedures vary, the general aim of these tests is to gauge the degree of association people have between pleasantness/unpleasantness and the concept of *physical activity* based on timing and accuracy of responses to stimuli (e.g., words) that represent these concepts.

The validity of these response latency tasks is, in part, dependent on how well the stimuli represent the targeted constructs (i.e., *physical activity* and *pleasantness*). For example, Bluemke et al. (2010) showed that automatic evaluations were more linked to behavior when the *pleasant* stimuli were words describing positive experiences with physical activity (e.g., ‘athletic’), as opposed to just pleasantness in general (e.g., ‘patient’). Additionally, to be generalizable across a broad range of study samples, the stimuli should be words that most people in a population tend to associate with the targeted concepts. This study will be the first to provide population-level evidence about the words that most people perceive as representative of physical activity and exercise behaviors and of the pleasantness associated with physical activity/exercise.

In addition to providing stimuli for measures of automatic evaluations of physical activity, the findings of this study might act also as a resource for tools to enhance automatic evaluations of physical activity. For example, *evaluative conditioning* works to enhance people’s automatic evaluations through repeated presentation of stimuli representative of the targeted behavior alongside pleasant stimuli (Hofmann et al., 2010). This technique is commonly applied in advertising and political campaigns (e.g., pairing sexually appealing images with soda products, or constantly using negative words paired with opposing political candidate names) and used in interventions that have effectively changed other health behaviors including alcohol consumption (Houben et al., 2010) and healthy eating (Hollands et al., 2011). The findings of this study, therefore, will assist in the development of tools to promote physical activity via enhancement of automatic evaluations.

Although the discussion to this point has focused on physical activity, it is possible that researchers may also wish to investigate ‘exercise,’ as opposed to physical activity. Physical activity and exercise have similar but distinct meanings in the public health literature (Caspersen et al., 1985). In accordance with the research literature, *exercise* is a specific goal-directed type of *physical activity*; however, it is unclear whether the general population distinguishes between the terms ‘physical activity’ and ‘exercise,’ as many people use them interchangeably. This is an important unanswered question that has implications to consider for developing physical activity interventions including, but not exclusive to, strategies targeting automatic evaluations like evaluative conditioning. As such, the primary aim of this study was to establish a set of words that adults perceive as strongly representative of the concepts of *physical activity* and *pleasant*, and the secondary aim was to determine whether Australian adults differentiate between behavior and pleasant words to describe *exercise* versus *physical activity*. It is hypothesized that the differences are mainly within the academic community, so there will be few differences at the population level.

## Materials and Methods

### Participants and Procedures

This study was a part of the 2015 National Social Survey – a population survey targeted at a random sample of Australian residents. Mobile and landline telephone numbers were dialed by a team of 34 interviewers via computer-assisted telephone interviewing by the Central Queensland University Population Research Laboratory in July – August of 2015. Gender-based and geographically proportionate random sampling of phone numbers was used to get near-equal sampling of men and women and to cover each state and territory area of Australia. Respondents were asked to participant in the study if they confirmed that they were 18 years of age or older. Participants (*N* = 1,318) were randomly assigned, in a ratio of 1:1 as determined by a computer-generated allocation, to answer the questions about either descriptors of ‘physical activity’ (*n* = 686) or ‘exercise’ (*n* = 632) behavior and pleasantness. A between-person design, as opposed to a within-person design in which the same participants were asked about ‘exercise’ and physical activity,’ was used to reduce the risk of response biases. Asking people to report words relevant to the term ‘physical activity’ and then to the term ‘exercise’ (or vice versa) may have resulted in people feeling pressured to come up with different word choices, even if they did not perceive a true difference between the two terms.

### Measures

A random half of the sample was asked, “Can you tell me five activities that you think about when you think of physical activity?” Interviewers clarified that, “We are looking specifically for behaviors, rather than feelings associated with physical activity.” Next, these participants were asked, “Can you tell me five words that you would use to describe a physical activity that you enjoy or find pleasant?” At this point, interviewers clarified, “We are looking specifically for feelings, rather than behaviors associated with physical activity.” The other half of the sample were asked the same questions about ‘exercise’ instead of ‘physical activity.’ Interviewers all used the same wording for each participant and recorded the participants’ immediate responses in the order that participants reported them.

### Data Coding and Analyses

Data were coded twice by independent reviewers (ALR, SS, SA) to assess (1) whether each response represented a novel ‘word’ category (*yes*/*no*), and (2) which ‘word’ category it represented (e.g., *aerobics, walking, calm*, and *competition*). Word categories represented responses with either the same stem (e.g., *walk* and *walking*) or the same meaning (e.g., *accomplishment* and *achievement*). There was no a priori determination as to what the ‘word’ categories would be or how many there would be. Interrater reliability was calculated as percentage agreement with a zero tolerance and unweighted Cohen’s Kappa (κ; Gamer et al., 2012; Gwet, 2014), with 0.40 < κ < 0.59 representing a weak level of agreement, 0.60 < κ < 0.79 representing moderate agreement, and κ ≥ 0.80 representing strong agreement (McHugh, 2012). Following the initial coding and reliability calculation, coding discrepancies were discussed amongst all three coders and the coding scores were adjusted accordingly to reflect the consensus code.

Scores were then calculated for each ‘word’ category as the number of participants that mentioned it, weighted by the ranking each participant gave it. The weighing was based on the assumption that people would report their most accessible or salient words first. Specifically, five points were given to a ‘word’ category for each time a person mentioned the ‘word’ first, four points for each mention as the second word, three points for each mention as the third word, two points for each mention as the fourth word, and one point for each mention as the fifth word. So, a score of 20 might represent a ‘word’ that four people mentioned as the first word representing the category or that 20 people mentioned as the fifth word representing the category. Based on the drop-offs of the distributions of the scores, it was determined that the top 20 ranked ‘words’ captured a reasonable sample of the most common responses.

## Results

The top 20 ranked words representing physical activity and exercise behaviors are presented in **Table 1** and the top 20 ranked words representing pleasant experiences of physical activity and exercise are presented in **Table 2**. Overall, there were not substantial differences between the words people used to describe physical activity and exercise.

**Table 1:** Rank ordering of the top 20 words people from independent samples used to describe physical activity or exercise behaviors and their associated representative scores.

Rank	Physical activity behavior words	Represen tative scores	Exercise behavior words	Represen tative scores
1	Walking	2027	Walking	1947
2	Running	1117	Running	1073
3	Swimming	795	Swimming	851
4	Gardening	763	Bike Riding	835
5	Bike riding	648	Gardening	440
6	Housework	482	Gym work outs	390
7	Gym work outs	451	Housework	301
8	Tennis	290	Tennis	279
9	Playing sport	282	Weight lifting	204
10	Manual labor	217	Playing sport	155
11	Golf	189	Golf	146
12	Football	186	Football	138
13	Weight lifting	150	Dancing	121
14	Yoga	116	Yoga	119
15	Dancing	115	Stretching	87
16	Farm work	110	Exercise machines	86
17	Chopping wood	98	Manual labor	85
18	Exercise	93	Exercise classes	84
19	Soccer	79	Playing with children	84
20	Hiking	73	Aerobics	78

**Table 2:** Rank ordering of the top 20 words people from independent samples used to describe pleasant experiences of physical activity or exercise behaviors and their associated representative scores.

Rank	Pleasant physical activity words	Represen tative scores	Pleasant exercise words	Represen tative scores
1	Relaxing	836	Relaxing	713
2	Happiness	573	Happiness	509
3	Enjoyment	571	Exhilarating	412
4	Exhilarating	484	Exhaustion	379
5	Exhaustion	452	Good	353
6	Good	350	Enjoyment	308
7	Energetic	323	Fun	298
8	Fun	301	Social	271
9	Refreshing	287	Healthy	271
10	Satisfying	278	Energetic	250
11	Social	260	Calm	245
12	Achievement	246	Refreshing	235
13	Healthy	233	Satisfying	222
14	Calm	228	Achievement	212
15	Pleasant	211	Invigorating	204
16	Beautiful	176	Pleasant	177
17	Invigorating	147	Challenging	172
18	Challenging	137	Fit	170
19	Clarity	136	Beautiful	107
20	Painful	130	Free	100

### Sample Characteristics

Response rate of the survey was 33%, which is typical for phone-based surveys (Curtin et al., 2000, 2005). There was a near equal sampling of gender (*n* = 716 women, 54.3%). Nearly half of participants were 55 years or older (*n* = 645, 48.9%), 16.6% (*n* = 219) were between the ages of 45–54 years, 14.1% (*n* = 186) were 35–44 years, and 19.3% (*n* = 255) were 18–34 years. A third of participants had education levels of secondary/high school or lower (*n* = 436, 33%), 22.2% had technical education or higher, and 44.2% (*n* = 583) had University or higher levels of education. In regards to employment status, 37.6% (*n* = 496) were employed full-time, 21.0% (*n* = 276) were employed part-time or casually, and the rest were unemployed (*n* = 61, 4.6%), retired or pensioners (*n* = 384, 29.2%), students (*n* = 35, 2.7%) or responsible solely for home duties (*n* = 57, 4.3%).

### Intercoder Reliability

Intercoder reliability ranged between moderate and strong. Reliability was highest for coding of whether or not each behavior word represented novel physical activity/exercise ‘word’ categories (physical activity behavior words: agreement = 95.9 to 97.8%; κ = 0.69 to 0.82; exercise behavior words: agreement = 96.4 to 97.3%; κ = 0.56 to 0.82). Reliability for whether or not each pleasant descriptor word was a novel ‘word’ category was acceptable (physical activity pleasant descriptors: agreement = 70.7 to 81.1%; κ = 0.70 to 0.79; exercise pleasant descriptors: agreement = 50.1 to 59.5%; κ = 0.47 to 0.53). Reliability for the ‘word’ categories of the physical activity/exercise behavior words remained within the strong/moderate range (physical activity behaviors: agreement = 79.6 to 89.8%; κ = 0.79 to 0.88; exercise behaviors: agreement = 77.7 to 82.8%; κ = 0.76 to 0.81). Reliability for the ‘word’ categories of the pleasant descriptors of physical activity/exercise was also within the acceptable range (physical activity pleasant descriptors: agreement = 70.8 to 81.2%; κ = 0.70 to 0.79; exercise pleasant descriptors: agreement = 50.1 to 64.6%; κ = 0.48 to 0.55). Lower reliability was commonly the result of one coding difference that occurred repeatedly amongst the most common answers.

### Physical Activity and Exercise Behaviors

The most common word used to describe physical activity and exercise behavior was *walking*. Following in popularity were *running, swimming, bike riding*, and *gardening* for both physical activity and exercise. Of note, people reported leisure activities such as *golf, dancing*, and *yoga* as well as activities like *housework* and *manual labor* in their responses for both physical activity and exercise. The only major difference in the words people used to describe physical activity and exercise was that *exercise machines* and *exercise classes* were commonly reported to represent exercise, but few people responded that these behaviors represented physical activity (exercise representative scores: *exercise machines* = 86, *exercise classes* = 84; physical activity representative scores: *exercise machines* = 6, *exercise classes* = 17).

### Pleasant Physical Activity and Exercise Descriptors

People described their pleasant experiences with physical activity and exercise similarly. People reported the words *relaxing, happiness, good, enjoyment, exhilarating*, and *exhaustion* as most representative. Some adjectives described mental states such as *clarity* and *energetic*, some focused on physical descriptors such as *healthy* and *fit*, and some focused on describing the activity such as *fun* and *challenging.* The words *clarity* and *painful* were reported as more representative as descriptors of physical activity (physical activity representative scores: *clarity* = 136, *painful* = 130) than of exercise (exercise representative scores: *clarity* = 72, *painful* = 74).

## Discussion

Researchers are beginning to measure and intervene with people’s automatic evaluations of physical activity (Rebar et al., 2016). This study provides empirically based word stimuli representative of *physical activity/exercise* behaviors and *pleasant* descriptors of physical activity/exercise for use in such research. Not surprisingly, the most common words people used to describe physical activity behaviors were in line with findings of previous survey research on people’s preferences for physical activities (Booth et al., 1997). The most common behavior words including transport (e.g., *walking*), leisure (e.g., *swimming*), and occupational (e.g., *manual labor*) activities. Almost all of the activities were aerobic. Generally, the stimuli used in previous studies of automatic evaluations of physical activity/exercise (e.g., Calitri et al., 2009; Scott et al., 2009; Conroy et al., 2010; Hyde et al., 2010; Rebar et al., 2015a) included more resistance-based (e.g., *lifting, sit-ups*) and fewer lifestyle (e.g., *gardening, manual labor*) behaviors than are present in the stimuli list from the present study. It may be that by not including certain types of physical activity in stimuli sets, these measures may not have fully captured automatic evaluations of the physical activity behaviors most relevant to certain individuals. Although the impact that the stimuli have on response latency measures is not clear, developers of such tasks suggest that the stimuli set should be well-representative and broad enough to encompass the entire targeted concept (De Houwer, 2001; Nosek et al., 2005). Researchers should consider incorporating stimuli that fully represent the relevant targeted behavior.

The pleasant descriptors of physical activity included pleasant-activated feelings (e.g., *exhilarating* and *energetic*) as well as pleasant-deactivated feelings (e.g., *relaxing* and *calming*). Some words described the instrumental value of physical activity (e.g., *healthy* and *fit*) and some words described more affective values (e.g., *fun* and *enjoyment*). This suggests people based their descriptions of pleasantness on both affective and instrumental attitudes, although evidence suggests that affective attitudes may be more predictive of physical activity behavior (Lowe et al., 2002; Rhodes et al., 2009). Most studies testing automatic evaluations of physical activity used generic positive/negative words and so were not similar to the stimuli produced from the present study (Calitri et al., 2009; Conroy et al., 2010; Hyde et al., 2010; Berry et al., 2011; Rebar et al., 2015a), but see Bluemke et al. (2010) and Brand and Schweizer (2015) for activity-based stimuli in German. The findings of Bluemke et al. (2010) suggest measures of automatic evaluations may be more linked to physical activity behavior if the adjective stimuli used are activity-related words; the outcomes of the present study make those types of stimuli more readily available for future researchers wishing to measure automatic evaluations.

There are a variety of measurement tools researchers can use to assess automatic evaluations including the Implicit Association Test (or variations thereof; Greenwald et al., 1998; Karpinski and Steinman, 2006; Siram and Greenwald, 2009), the evaluative priming method (Fazio et al., 1995; Eves et al., 2007), or the impulsive approach and avoidance manikin task (Krieglmeyer and Deutsch, 2010). The stimuli that emerged in this study can also be a resource for the assessment of other non-conscious constructs beyond automatic evaluations. For example, the list of physical activity or exercise words can be utilized when testing automatic associations between *physical activity/exercise* and *self* as a measure of non-conscious self-schema (e.g., Banting et al., 2009). Alternatively, the *physical activity/exercise* stimuli might be utilized in measures of attention biases such as via the dot probe task (e.g., Calitri et al., 2009). The present study list of stimuli will also likely be applicable for measures of self-reported affective and instrumental evaluations of physical activity.

Beyond measurement, these stimuli can be a resource for novel evaluative conditioning intervention tools that are integrated in broader health behavior interventions, as there has been a call to incorporate more strategies to target non-conscious regulation (Marteau et al., 2012; Sheeran et al., 2013). Evaluative conditioning has demonstrated long-lasting effects on behavior (De Houwer et al., 2001). Indeed, many people can attest to these long-term consequences when they have an automatic disgust response as a result of a long ago learned association of a particular food with nausea or when distant memories are provoked by a certain odor. Harnessing these long-term conditioning effects has potential for enhancing the effectiveness of physical activity interventions. Evaluative conditioning is only one strategy for intervening with automatic evaluations and other possibilities likely exist. For example, it may be that just by highlighted the positive attributes of physical activity in physical activity interventions (e.g., ‘*isn’t this fun?,” “wasn’t that relaxing?”*), people will be more likely to maintain regular activity because of the powerful motivational influence of recalling pleasant aspects of the experience (Kwan and Bryan, 2010). This study provides word stimuli resources to be used within such studies.

In addition to providing the stimuli list as a resources for future research, this study demonstrated that the words Australian adults use to describe *physical activity* are not substantially distinct from those used to describe *exercise*. This suggests that the distinction typically made in research that exercise is a goal-directed type of physical activity (Caspersen et al., 1985) may not be made by the general population. Some studies have focused on automatic evaluations of *physical activity* (e.g., Conroy et al., 2010; Hyde et al., 2012; Rebar et al., 2015a), whereas some have focused on *exercise* more specifically (e.g., Berry and Spence, 2009; Calitri et al., 2009; Bluemke et al., 2010). In light of the present findings, it may be the case that these measures of automatic evaluations of physical activity/exercise are targeting the same constructs. This finding points toward the possibility of summative work across these studies, although such efforts will be largely dependent on consistency of the behavioral measures.

### Study Limitations

This study was designed to be representative of an Australian adult population. However, compared to the national population, the sample is older, on average (Australian Bureau of Statistics, 2013). As such, the findings cannot be generalized as being representative of all Australian adults. For example, the older population may explain why lifestyle activities were more commonly reported than resistance training activities. The set of words were meant to represent generic perceptions and are not tailored for specific population subsamples. Additionally, given that this study was conducted to obtain population-level evidence, all the behavior and pleasant descriptor words may not be applicable at an individual-level. Adjustments of the stimuli may be necessary for use in specific populations (e.g., men or women, specific age groups, people with specific chronic conditions). Alternatively, researchers may wish to design options for tailoring tests in an idiosyncratic fashion, so that the stimuli used are applicable for each person. Although the words seem broadly generalizable for English-speaking populations, this study sample was Australian and, therefore, the representativeness of the words may not be generalizable to people in other countries.

Another limitation is that the word stimuli lists were developed through self-reported methods, so might be more representative of deliberative, reflective evaluations and may not fully represent automatic evaluations. However, by asking participants to spontaneously report the words (without much deliberation), we have taken efforts to try and capture people’s automatic responses. Additionally, although there was good intercoder reliability, by nature the coding of words as having similar meanings is a subjective task, and therefore, it cannot be ruled out that judgments made by the coders misrepresent the true meaning of the respondents on occasion.

Finally, although this study provides an important resource for use within response latency measures, the word lists produced may not meet all the stimuli needs of these measures, and therefore researchers may need to seek other resources to find other stimuli (e.g., general positive/negative word stimuli are available; see Bradley and Lang, 1999). Population-based evidence of negative descriptors of physical activity is not yet available; therefore researchers seeking to examine negative associations may need to conduct some pilot testing or base their stimuli on previously tested stimuli (e.g., Bluemke et al., 2010).

## Conclusion

Automatic evaluations are underutilized in investigations of the psychology of physical activity and as a tool for increasing people’s physical activity levels. This study provides population-level evidence-based sets of words that are highly representative of *physical activity/exercise* and *pleasant* descriptors of physical activity/exercise. These words can be used as a resource in efforts to better measure automatic evaluations in response latency tasks and to enhance automatic evaluations via evaluative conditioning tasks. The next step in this line of research is to find effective ways to utilize this resource to increase physical activity levels and stimulate the physical health benefits with which physical activity is associated (Warburton et al., 2006; Sattelmair et al., 2011; Rebar et al., 2015b).

## Author Contributions

AR, SS, SA, and CV helped conceive of the idea of the study design, assisted in coding, analyzing the data, interpreted the findings, and provided intellectual content for manuscript. CS, JD, BJ, DC, and RR helped conceive of the idea of the study design, assisted in interpreting the findings, and provided intellectual content for manuscript.

## Conflict of Interest Statement

The authors declare that the research was conducted in the absence of any commercial or financial relationships that could be construed as a potential conflict of interest.
